# MXenes for Bioinspired Soft Actuators: Advancements in Angle-Independent Structural Colors and Beyond

**DOI:** 10.1007/s40820-024-01367-8

**Published:** 2024-03-04

**Authors:** Siavash Iravani, Rajender S. Varma

**Affiliations:** 1 Independent Researcher, W Nazar ST, Boostan Ave, Isfahan, Iran; 2 Centre of Excellence for Research in Sustainable Chemistry, Department of Chemistry, Federal University of São Carlos, São Carlos, SP 13565-905 Brazil

**Keywords:** MXenes, MXene-based composites, Bioinspired soft robotics, Angle-independent structural color

## Abstract

Soft actuators have garnered substantial attention in current years in view of their potential appliances in diverse domains like robotics, biomedical devices, and biomimetic systems. These actuators mimic the natural movements of living organisms, aiming to attain  enhanced flexibility, adaptability, and versatility. On the other hand, angle-independent structural color has been achieved through innovative design strategies and engineering approaches. By carefully controlling the size, shape, and arrangement of nanostructures, researchers have been able to create materials exhibiting consistent colors regardless of the viewing angle. One promising class of materials that holds great potential for bioinspired soft actuators is MXenes in view of their exceptional mechanical, electrical, and optical properties. The integration of MXenes for bioinspired soft actuators with angle-independent structural color offers exciting possibilities. Overcoming material compatibility issues, improving color reproducibility, scalability, durability, power supply efficiency, and cost-effectiveness will play vital roles in advancing these technologies. This perspective appraises the development of bioinspired MXene-centered soft actuators with angle-independent structural color in soft robotics.

## Highlights

- MXene-based soft actuators with angle-independent structural colors have the potential to contribute to various fields, including display technologies, camouflage systems, sensors, and beyond.
- Bioinspiration has paved the way for developing advanced structural colored soft actuators for biomimetic soft robots.
- This perspective appraises the development of bioinspired MXene-based soft actuators with angle-independent structural color and beyond in soft robotics.

## Graphical abstract

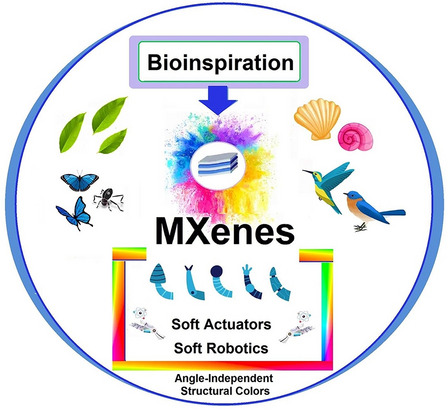

## Introduction

Structural coloration found in nature has fascinated scientists for a long time, as various living organisms possess the ability to display vibrant and iridescent colors without the deployment of pigments [1–3]. This phenomenon can be achieved through intricate nanostructures that manipulate light at the microscopic level. Indeed, structural color, which arises from the interaction of light with nanostructured materials, has captivated scientists and researchers for its fascinating optical properties. Traditional structural color typically exhibits angle-dependent effects, meaning that the perceived color changes with the viewing angle. However, recent advancements in the field have focused on developing angle-independent structural color, offering exciting opportunities for practical applications. Mimicking such angle-independent structural color in synthetic materials has been a challenging task, but recent innovations in MXene-based soft actuators have shown promising results [4]. One challenge in developing angle-independent structural color lies in achieving a broad range of colors; traditionally, they often rely on interference effects, resulting in limited color options. However, novel approaches such as photonic crystals, plasmonic structures, and metamaterials have shown promise in expanding the color gamut, enabling the creation of vibrant and diverse angle-independent structural colors [5–8].

MXenes are a family of two-dimensional (2D) transition metal carbides, nitrides, and carbonitrides that possess unique mechanical, thermal, optical, magnetic, and electrical properties [9–11]. They have reaped momentous consideration in the area of soft robotics due to their excellent flexibility, high conductivity, and tunable properties [12–14]. The distinctive amalgamation of mechanical strength, electrical conductivity, sensing capabilities, and biocompatibility make MXenes a promising material choice for various soft robotic applications. MXenes can be integrated into soft robotic systems to enable electrical conductivity and facilitate sensing capabilities, allowing for more precise control and feedback in the robot's movements and interactions. Notably, MXenes can enhance the durability and mechanical properties of soft robotic materials [15]. By incorporating MXenes into the structure or as coatings, the ensuing materials exhibit improved strength, toughness, and resistance to wear and tear [16]. MXenes can improve the toughness and wear resistance of materials by enhancing their mechanical strength, thermal stability, wear resistance, and electrical conductivity. These properties render MXenes an attractive choice for incorporation into various materials, leading to the development of high-performance, durable, and wear-resistant products [17, 18]. For instance, Ti_3_C_2_T_x_ MXene multilayer coatings were constructed with superior wear resistance properties by Grützmacher et al. [19]. MXene coatings possess excellent lubrication and anti-friction properties, thus reducing the friction and wear between surfaces in contact [20, 21]. MXene nanosheets have emerged as a remarkable accomplishment in the realm of 2D materials as solid lubricant coatings. Their unique characteristic lies in their capacity to form tribo-films exhibiting both low-friction and wear-resistant properties, an exceptional feature greatly enhancing their durability and resistance to wear [22]. Indeed, the unique layered structure of MXenes allows for easy shear and sliding, minimizing surface damage and enhancing the material's ability to resist wear and tear [20, 21].

Bioinspired MXene-centered soft actuators offer exciting opportunities for producing advanced soft robotic systems that can mimic and surpass the capabilities of their natural counterparts. For instance, an innovative soft tubular actuator, inspired by phototropism, has been developed using MXene reinforcement. This actuator exhibited the remarkable capability of self-orienting in all directions by swiftly responding to incident light from any angle in a three-dimensional (3D) space. It also exhibited omnidirectional sensing, continuous tracking, and adaptive interaction with light, making it a highly versatile and responsive system [23]. Since the field of MXenes in soft robotics and actuators is until now relatively new, additional explorations are warranted to explore their maximum potential [24–26]. Meanwhile, important aspects such as scalability, long-term stability, and integration with other soft robotic components need to be addressed for the widespread adoption of MXenes in this domain. Nonetheless, the unique properties of MXenes provide them a promising avenue for advancing the capabilities of soft robotics [14, 27].

Overall, structural colors have garnered noteworthy consideration lately due to their unique optical properties and potential applications in various fields. MXene-based soft actuators, a burgeoning area of research, hold great promise in developing dynamic and responsive materials [14] as they represent a novel approach in the advancement of biomimetic multifunctional materials with enormous promise for diverse applications. These include the development of soft walkers, grippers, and fluttering wings [15, 25, 28, 29]. Angle-independent structural colors refer to colors that remain consistent regardless of the viewing angle. This property is highly desirable as it allows for more reliable and consistent coloration in applications such as display technologies, camouflage systems, and sensors. Implementing angle-independent structural colors in MXene-based soft actuators presents several key advantages and challenges [30]. The importance of angle-independent structural colors lies in their ability to provide accurate and reliable coloration. In applications where color accuracy is crucial, like displays or sensors, angle-independent structural colors ensure consistent color perception from different viewing angles. This improves the overall user experience and enhances the functionality of devices utilizing these materials [31]. One significant obstacle is the fabrication of structures that can control and manipulate light at various angles. MXene-based soft actuators typically rely on the arrangement and orientation of nanostructures to achieve coloration. Designing and engineering these structures to maintain color integrity across all angles requires precise control over their size, shape, and arrangement. In addition, the optimization of mechanical properties alongside angle-independent coloration is very important. Soft actuators based on MXene materials often require flexibility and stretchability to enable their desired functionality. Balancing the mechanical properties of these materials while ensuring angle-independent structural colors can be a complex task, requiring careful consideration of material composition and design. Furthermore, the limited availability of data and published articles in the field of MXene-based soft actuators with angle-independent structural colors adds to the challenges. The infancy stage of research in this area means that there is a vital demand for more comprehensive studies and experimental data to guide the future development and implementation of these materials. By examining the existing literature, this perspective aims to highlight key advancements, challenges, future prospects, and trends in this emerging field.

## Bioinspired Materials with Angle-independent Structural Colors

Nature has long been the greatest inspiration for scientists when it comes to developing new materials [32–35]. Many organisms in the natural world, such as butterflies, beetles, and birds, possess remarkable structural colors that remain vivid regardless of the observer's viewpoint. These colors arise from the unique nanostructures found in their scales, feathers, or exoskeletons. Various bioinspired materials have been constructed with angle-independent structural colors [36–38] as they enjoy numerous advantages when compared to angle-dependent counterparts. They exhibit consistent colors, irrespective of the viewing angle, similar to dyed materials. However, they possess an additional advantage of being highly resistant to fading [31]. Besides, these colors offer a wider range of tunability, making them extremely versatile for numerous applications [31]. The ability to maintain color consistency across various viewing angles is a significant advantage as it renders these materials suitable for appliances where color perception remains consistent, regardless of the observer's viewpoint. In addition, angle-independent structural colors find utility in semitransparent organic solar cells, effectively demonstrating viewing angle-independent Janus structural colors [39]. This characteristic opens up significant opportunities for their utilization in building-integrated photovoltaics and portable electronic devices [39]. The combination of these advantages makes angle-independent structural colors highly desirable for an extensive range of applications, encompassing consumer products and energy devices.

Nanostructures play a pivotal role in producing angle-independent structural colors as they can be patterned in various ways, including periodic lattices, multilayers, or hierarchical structures. The size and spacing of these nanostructures determine the wavelengths of light that are selectively reflected or scattered, resulting in the perception of specific colors. In one study, inspired from the anisotropic lattice microstructure of butterfly, Zhang et al. [36] developed a unique angle-independent structural entity. They achieved this by introducing spinous pollen particles into colloidal crystal arrays, disrupting their self-assembling procedure. As a result, the composite materials exhibited anisotropic close-packed colloidal crystal domains surrounding the pollen spikes. These domains positioned in different directions enabled the reflection of light at a broad scope of viewing angles, bestowing the composites their angle-independent structural colors. Furthermore, they introduced photothermal responsive graphene-tagged hydrogels to create light-regulated reversible structural color altering conduct in the materials. These innovative bioinspired angle-independent structural color entities hold great promise in various applications, including the construction of intelligent sensor and anti-counterfeiting bar code label [36]. In another study, self-restoring organogel nanocomposites were developed, featuring angle-independent structural colors [40]. This approach involved the co-assemblage of carbon black, oleophilic nanoparticles of silica, and silicone-based supramolecular gels. By utilizing this organogel arrangement, amorphous accumulation of silica nanoparticles could be achieved, leading to the generation of angle-independent structural colors within the produced nanocomposite. Besides, the presence of hydrogen bonding in the supramolecular gel imparted self-restoring capabilities to the organization. Consequently, the ensued structural colored films demonstrated the ability to rapidly self-heal within a matter of seconds, restoring surface slipperiness, storage modulus, and structural color even after experiencing mechanical cuts or multiple shear failures [40].

While the development of bioinspired materials with angle-independent structural colors has shown promising results, there are still several challenges that need to be addressed. One of the obstacles lies in replicating the intricate and complex nanostructures found in nature. Achieving precise control over the size, shape, and arrangement of these nanostructures is crucial for accurately reproducing the vibrant colors observed in natural organisms. Besides, scaling up the production of these bioinspired materials in a cost-effective manner ought to be considered. The manufacturing processes need to be optimized to achieve large-scale production and commercial viability. Notably, ensuring the long-term durability and stability of these materials is crucial for their practical appliances, withstanding environmental components such as moisture, temperature changes, and UV radiation without significant degradation. Despite these limitations, the field of bioinspired materials with angle-independent structural colors holds immense potential for the future. The next-generation screens can be envisioned that produce vibrant colors without the need for traditional backlighting, leading to energy savings and improved visual experiences. Moreover, smart sensors and imaging devices can be developed and integrated into optical systems, creating sensors to detect specific wavelengths or changes in environmental conditions. Importantly, the unique and difficult-to-replicate structural colors can be utilized in the creation of anti-counterfeiting bar code labels and other security features. These materials could help prevent forgery and protect valuable products from unauthorized replication.

## Recent Advancements

All-embracing investigation has been devoted to the advancement of artificial structurally colored soft actuators, aiming to replicate natural color functionality and programmable shape alterations in retort to external stimuli. To achieve this, scientists have explored various approaches inspired by biological systems [41]. For instance, humidity-pushed structurally colored soft actuators have been devised with the ability to switch between multiple colors by incorporating chromogenic photonic crystals into flexible materials. This concept draws inspiration from the molecular channels found in living cells and tissues [41]. Similarly, vapor-propelled structurally colored soft actuators have been acquired by integrating patterned polymer strips into synthetic inverse opal films [42]. These actuators imitate the adaptive color adjustment mechanism observed in chameleons, thus enabling programmable shape transformations and biomimetic color changes [42]. In comparison with chemically colored soft actuators that rely on pigments/dyes, structurally colored soft actuators unified with photonic crystals offer dynamically tunable dazzling colors that do not fade provided that the periodic structures endure [30]. Yet, a limitation of the currently reported structural color of soft actuators is their dependence on viewing and light radiance angles, which sets them apart from chemically colored soft actuators. It is worth noticing that the growth of angle-independent structural colors in soft actuators, resembling pigments and dyes then again without the requirement to absorb light, would be highly valuable for industrial applications. Envisioning the creation of structurally colored soft actuators capable of maintaining a consistent color when observed from any angle under sunlight as reflecting light would be a significant advancement [30].

One of the key advantages of soft actuators is their ability to produce complex and intricate movements, similar to those found in biological systems. This flexibility renders them appropriate for a broad range of appliances, including soft robotics, wearable gadgets, medical devices, and human–machine interfaces [43, 44]. On the other hand, structural colors, which arise from the interaction of light with nanostructures rather than pigments, have garnered considerable consideration lately in view of their potential applications in assorted arenas, comprising displays, sensors, and camouflage [6, 45, 46]. One area of particular interest is the implementation of structural colors in MXene-based soft actuators. For instance, creating camouflage matters that can adaptively alter color in both the infrared (IR) and visible regions is a fascinating yet demanding endeavor [47]. Zhang et al. [47] developed a novel approach to fabricate dynamic camouflage materials by growing photopolymerizable blue phase liquid crystals onto very well-aligned MXene nanostructured thin films. The ensuing 3D soft photonic crystals, integrated with MXene, exhibited vibrant structural colors and could switch among a dark black state and a bright colored state up on applying a low DC electric field. They demonstrated proof-of-conception pixelated contrivances that enabled pixel-controllable electrochromism. Besides, a flexible film consisting of these 3D soft photonic crystals was constructed, providing thermal camouflage and visible electrochromism by utilizing the exceptional electrothermal conversion as well as low mid-IR emissivity of MXene nanomaterials [47].

Smart soft actuators with closed-loop control, self-adaptive changes in color, shape, and stiffness hold great appeal across a wide range of applications, including energy harvesting, cargo manipulation, medical treatment, and motion monitoring [48, 49]. For instance, an insect larvae enthused MXene-centered soft actuators were developed, which were capable of changeable jumping movement regulated by light [50]. These actuators effectively and safely interact with the environment, providing superior performance. The utilization of smart materials, such as electrically, thermally, magnetically, and photoresponsively actuated materials, empowers soft structures to realize programmable shape-morphing capabilities and observe environmental variations. Several review articles have focused on the benefits of miniaturization and untethered actuation, positioning magnetically responsive actuators as the ideal choice for robotics applications [48, 51]. Furthermore, they emphasize the significance of novel materials, structural designs, and actuation methods in engineering adaptable, multimodal propulsion-enabled, self-restoring, and multi-responsive soft actuators. They also shed light on the real-life appliances of these actuators, as well as the limitations and prospects that lie ahead for next-generation soft actuator development [51–53]. Soft actuators with adaptive properties offer a versatile solution for handling and manipulating objects of diverse sizes and weights; they find applicability in a range of tasks, including sorting, transporting, and assembling objects [51]. Notably, soft actuators possess the remarkable capability to alter their shape and stiffness, which can be effectively utilized to harness power from various environmental sources. These sources include sunlight, wind, or even the actuator's own deformation, enabling the generation of sustainable energy. These actuators with self-adaptive properties offer the potential to monitor and control the motion of objects or systems, delivering valuable insights for a wide range of purposes. These applications include but are not limited to rehabilitation devices, prosthetics, wearable devices, and robotics [51, 52, 54].

**Fig. 1:**
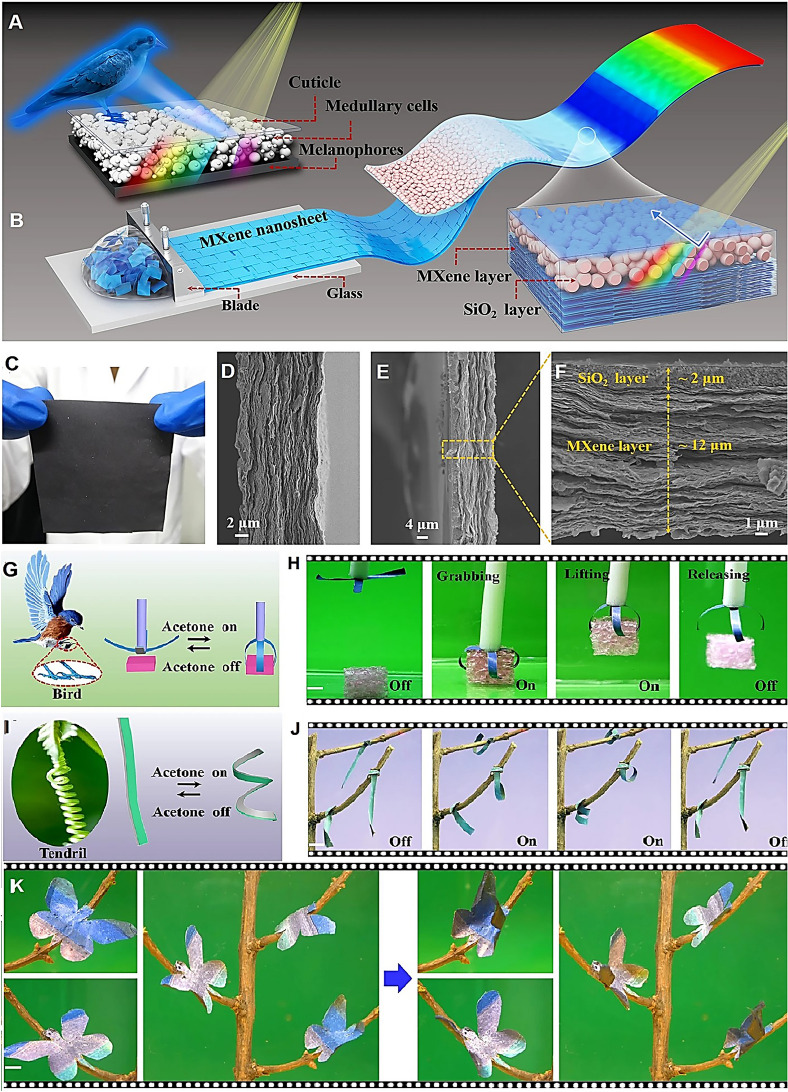
Bioinspired MXene-based soft actuators with their angle-independent structural color: **A** The plumage of the plum-throated cotinga exhibits angle-independent structural color in nature. **B** The process of fabricating MXene-centered soft actuators with angle-independent structural color is shown schematically. **C** A very well-aligned MXene film was created deploying a blade coating procedure, as seen in the digital photograph. **D** The cross-sectional scanning electron microscopy (SEM) image shows the structure of the fabricated MXene film. **E, F** Cross-sectional SEM images of biomimetic MXene-centered soft actuators are shown at low and high magnifications. **G, H** A blue gripper is designed with angle-independent structural color, enthused by the bird's claw. **I, J** Simulated tendrils are created with angle-independent green structural color, allowing them to twirl about tree twigs reversibly. **K** Imitation butterflies are developed with multiple angle-independent structural colors, and their wings flutter up and down on tree twigs upon cyclic exposure to vapors of acetone (scale bar: 0.5 cm). Reproduced with permission from ref [30]. Copyright 2022 Springer Nature, under the terms of the Creative Commons CC BY license

Scientists have drawn inspiration from various living organisms in nature that possess soft-bodied actuation distinctive and structural colors. This source of inspiration has paved the way for the development of innovative structural colored soft actuators for biomimetic soft robots [4, 13, 14]. However, a significant challenge arises when attempting to replicate both the angle-independent structural color and shape-morphing proficiencies of the plum-throated cotinga (*Cotinga maynana*) flying bird [30]. Impressively, Xue et al. [30] have introduced MXene-centered soft actuators that mimic the angle-independent structural color (Fig. 1). These actuators were created by self-assembling colloidal SiO_2_ nanoparticles onto very well-aligned MXene films, trailed by vacuum-aided infiltration of polyvinylidene fluoride into the interstices. The ensuing soft actuators exhibited angle-independent structural color, showing swift actuation and retrieval speediness. They could achieve a highest curvature of 0.52 mm^−1^ within 1.16 s and recover in ~ 0.24 s when exposed to acetone vapor. These structural colored soft actuators were employed to create a blue gripper-like bird's claw capable of capturing targets, artificial green tendrils that could twirl about tree branches, and an artificial multicolored butterfly that could flutter its wings cyclically when exposed to vapors of acetone. This tactic offered valuable insights for the growth of biomimetic multifunctional soft actuators, enabling advancements in somatosensory soft robotics and the next-generation intelligent machineries [30].

Overall, angle-independent structural colors offer a visually appealing aspect to soft actuators, as they offer a broader range of colors that remain consistent regardless of the viewing angle. This can greatly enhance the overall esthetic appeal of soft actuators, making them more attractive and engaging for users. Importantly, implementing angle-independent structural colors in MXene-based soft actuators can facilitate improved visibility and recognition in various applications [30]. For instance, in robotics or wearable devices, these colors can be deployed to indicate different states or modes, making it easier for users or observers to understand the device's status at a glance [2, 55]. Angle-independent structural colors can also have functional significance in certain appliances. For instance, in biomedical devices, these colors can be used to visualize and monitor changes in pressure, temperature, or chemical composition, enabling real-time feedback and diagnostics [56, 57]. However, there are some crucial aspects to address, including material compatibility, tunability, stability, and manufacturing scalability. MXenes offer unique mechanical properties, but may not inherently possess the required nanostructures for angle-independent structural colors. Overcoming this limitation requires careful selection and integration of suitable nanostructures within the MXene-centered soft actuators. Achieving angle-independent structural colors often requires precise control over the size, shape, and arrangement of nanostructures; this tunability is crucial to acquire the desired color properties. In addition, maintaining the stability of these structures in the dynamic and deformable environment of soft actuators poses a significant challenge. Ensuring the long-term stability and color retention will require innovative approaches in material design and fabrication techniques. Notably, to make bioinspired MXene-centered soft actuators with angle-independent structural colors commercially viable, it is essential to develop manufacturing processes that can be easily scaled while maintaining the desired color properties. This requires optimizing fabrication techniques, considering cost-effectiveness, and ensuring reproducibility on a large scale.

## Advantages and Disadvantages

Angle-independent structural color is a fascinating phenomenon which has garnered major consideration lately. In this context, MXene-based soft actuators have emerged as promising candidates for achieving angle-independent structural color. These MXene-centered soft actuators offer several advantages such as wide viewing angles, tunability, flexibility, and fast response times. However, they also have limitations, including a limited color range, sensitivity to environmental conditions, and manufacturing complexity.

### Advantages

*Wide viewing angle* one of the key advantages of MXene-based soft actuators with angle-independent structural color is their ability to exhibit structural color over a broad span of viewing positions. This characteristic renders them suitable for various applications, including displays, sensors, and camouflage systems.

*Tunability* MXene-based soft actuators offer tunability in terms of color and intensity. The choice of MXene material and its integration with other components allow for the precise control of structural color, enabling customization according to specific requirements.

*Flexibility* MXene-based soft actuators possess excellent flexibility, making them suitable for applications that require conformability to curved surfaces. This elasticity opens up opportunities for their use in wearable devices, flexible electronics, and biomedical applications.

*Fast response time* MXene-centered soft actuators exhibit a fast response time, enabling rapid color change in response to outside stimuli. This attribute is beneficial for applications such as dynamic displays, active camouflage, and responsive sensors.

*High mechanical compliance* MXene-centered soft actuators possess the ability to undergo substantial stretching and deformation, rendering them well suited for implementation in kinetic soft robotics and wearable devices [27, 58]. In a study conducted by Xue et al. [30], it was observed that these structurally colored soft actuators displayed impressive mechanical properties, including a tensile strength reaching 7 MPa [30]. In another study, the MXene/polyethylene-based soft actuators demonstrated quick and significant bending deformation when subjected to various external stimuli, including light, electricity, heat, and humidity [59]. Utilizing the straightforward fabrication process and the anisotropic, customizable, and programmable nature of these actuators, Xu et al. successfully constructed a light-responsive soft robot capable of directional crawling. Furthermore, leveraging the actuator's high sensitivity to light and humidity, a smart clothing prototype was developed, enabling reversible bending deformation in response to natural sunlight exposure and sweat conditions [59].

### Disadvantages

*Limited color range* While MXene-based soft actuators offer tunability, the available color range is still limited compared to traditional pigments or dyes. Thus, certain color requirements may not be achievable using MXene-based systems, which may restrict their application in certain fields.

*Sensitivity to environmental conditions* MXene materials can be sensitive to environmental factors such as humidity and temperature. This sensitivity may affect the stability and performance of angle-independent MXene-based soft actuators, necessitating careful consideration when designing and operating these systems.

*Scalability and manufacturing complexity* The fabrication process of MXene-centered soft actuators can be intricate and time-consuming. This complexity may pose challenges in scaling up production and commercialization, potentially limiting widespread adoption of this technology.

*Environmental impact* The environmental impact of MXene-based soft actuators and their associated manufacturing processes still requires further investigation. Assessing the sustainability of these materials and optimizing their synthesis methods will be crucial for minimizing any potential ecological footprint.

## Challenges and Future Perspectives

The development of bioinspired MXene-centered soft actuators exhibiting angle autonomous structural color not only expands our understanding of natural coloration mechanisms but also paves the way for the advancement of intelligent devices and somatosensory soft robotics of future. The combination of structural color and shape switching capabilities in these soft actuators opens up new opportunities for innovative usages in assorted arenas such as advanced robotics, adaptive disguise, and responsive displays [30]. However, challenges remain in replicating complex structures, scaling up the fabrication aspects, and addressing the complexity of control system. Despite these impediments, their soft and flexible nature, rapid actuation and recovery, and vibrant coloration provide exciting opportunities for biomimetic designs and advanced applications. Overcoming limitations such as environmental compatibility and material durability will be crucial for realizing the full potential of these innovative soft actuators [13, 14, 27]. More elaborative studies in this area can help to unlock their broader usage and impact in various industries.

One of the challenges researchers face pertain to the replication of intricate nanostructures found in nature that enable angle-independent structural color. Achieving the same level of complexity and precision in synthetic materials can be an arduous task [30]. In addition, as with many novel materials, a challenge lies in scaling up the manufacturing process of MXene-based soft actuators. Ensuring consistent quality and reproducibility on a larger scale is crucial for practical applications. Notably, operating and controlling MXene-based soft actuators can be complex. The development of control systems that accurately and efficiently activate and manipulate these actuators requires further research and exploration. The response and performance of MXene-based soft actuators may be affected by environmental factors, namely temperature, humidity, and chemical exposure. Understanding and mitigating the potential limitations imposed by these factors are crucial for the real-world applications. Consequently, the enduring resilience and stability of MXene-centered soft actuators ought to be investigated further as assurance of their reliability and robustness over extended periods of use is indispensable for practical applications and commercial viability [13, 14, 27]. Some of the main challenges are summarized below:

### Material Compatibility

Integrating MXenes with other materials to realize bioinspired soft actuators with angle-independent structural color can present compatibility issues. Ensuring proper bonding and compatibility between MXenes and other components is crucial to attain optimal performance and durability [60–63]. Surface modification techniques can be utilized to enhance the compatibility between MXenes and other materials. Functionalization of the MXene surface with appropriate chemical groups can improve its affinity toward different materials, thus facilitating bonding and compatibility.

### Color Reproducibility

Achieving consistent and reproducible colors across different devices and manufacturing processes is a challenge for angle-independent structural color. Variations in fabrication techniques, environmental factors, and material properties can impact color accuracy, thus rendering it important to develop standardization methods to ensure reliable color reproduction [30]. Indeed, one of the key challenges in color consistency is the variation in fabrication techniques. Different manufacturing processes and parameters can lead to variations in the thickness, composition, and arrangement of the MXene layers, thereby affecting the resulting color. To address this, standardization methods can be developed to establish instructions and protocols for the fabrication process. These guidelines can specify the optimal parameters and techniques to achieve consistent coloration, thus reducing variations across different devices and fabrication setups. In addition, environmental factors also play a significant role in color accuracy. Factors such as temperature, humidity, and lighting conditions can introduce variations in color perception. To mitigate these effects, standardized methods can include recommendations for controlled environments during fabrication and color assessment. This may entail maintaining specific temperature and humidity levels, as well as employing consistent lighting setups for color evaluation. The properties of MXene-based materials themselves can also contribute to color variations. Deviations in the MXene composition, layer thickness, or surface modifications can result in different color appearances. Standardization methods can address these challenges by specifying material requirements, such as specific MXene compositions or thickness ranges, to ensure consistent color reproduction. Notably, guidelines for characterizing and measuring color can be established to provide a standardized approach for color evaluation across different devices and manufacturing processes. The implementation of standardization methods not only helps achieve color consistency but also enables reproducibility. By establishing clear guidelines and protocols, manufacturers can ensure that the color performance of MXene-based soft actuators remains consistent across different production runs and devices, thus enhancing the reliability and acceptance of these actuators in practical applications.

### Scalability

The synthesis of MXenes can be a complex and time-consuming process, thus manufacturing large quantities while maintaining quality and consistency can be demanding [64–67]. The fabrication techniques utilized for soft actuators can change based on the particular type of actuator and the desired characteristics. A wide variety of manufacturing methods are commonly employed, such as laser cutting, electrowetting, 3D/4D printing, textile processing, electrospinning, origami folding, and casting/molding [68]. In this context, fabricating MXene-based soft actuators can be performed via several methods such as 4D printing, spin coating, and blade coating [25, 30, 58]. For instance, taking inspiration from the intricate design of bamboo, a novel soft actuator was developed with a hierarchical gradient structure. This innovative design incorporated the assembly of micro–nano-sized 2D MXenes and one-dimensional nanofibers of cellulose; the resulting structure was strengthened by molecular-scale strong hydrogen bonding [28]. The development of safer, scalable, and cost-effective manufacturing methods is essential to enable widespread approval. Streamlining and optimization of the fabrication process will be necessary to enable the pervasive acceptance of MXene-based soft actuators in biomedical and healthcare applications. To ensure the widespread acceptance of MXene-based soft actuators, it is crucial to establish robust quality assurance and control mechanisms. This involves implementing stringent testing protocols at various stages of the manufacturing process to validate the performance, reliability, and durability of the actuators.

### Durability and Longevity

Ensuring the durability and longevity of MXene-based soft actuators with angle-independent structural color is crucial for their practical appliances. These materials should be able to withstand repetitive motion, environmental conditions, and aging without degradation or loss of color performance; improvements in terms of the stability and robustness of MXenes are essential for long-term functionality. Repetitive motion poses a challenge to the durability of MXene-based soft actuators. These actuators are designed to undergo repeated cycles of expansion and contraction, which can put stress on the materials. To enhance their durability, it is crucial to develop MXene-based structures that can withstand such mechanical strain over an extended period. This entails optimizing the mechanical properties of the materials, such as their elasticity and resilience, to ensure they can withstand the repetitive motion without deterioration. Notably, environmental conditions play a significant role in the durability of MXene-based soft actuators as these actuators may be exposed to various factors such as temperature variations, humidity, and chemical exposure. To enhance their longevity, MXenes should be engineered to be resistant to these environmental factors. Developing coatings or protective layers shielding the MXene materials from adverse conditions can help maintain their structural integrity and color performance. Furthermore, aging is another factor that can impact the functionality of MXene-based soft actuators over time. Aging processes, such as oxidation or degradation, can affect the stability and performance of the materials. To improve the long-term functionality, MXenes should be engineered to be more resistant to aging effects. The optimization of the material composition, modification of surface properties, or introduction of additives can help enhance the stability, thus preventing possible degradation.

### Stability

MXenes are susceptible to oxidation under ambient conditions, which can degrade their properties over the time. Developing strategies to enhance their stability and prevent oxidation is crucial for long-term functionality. Some MXenes, particularly those exposed to harsh chemicals or extreme environments, may experience chemical degradation, affecting their stability and performance [69, 70]. In one study, MXene-based hydrogels have been constructed for bioinspired somatosensory soft actuators, showing strain sensitivity along with high steadiness after > 300 loading–unloading cycles at 100% strain [71]. Researchers are exploring surface modifications, protective coatings, or selecting MXene compositions that exhibit enhanced chemical stability to mitigate these challenges. The employment of suitable techniques for optimizing the synthesis and fabrication processes can result in MXene structures with improved chemical stability. Thermal stability is another crucial aspect, especially when MXene-based soft actuators are subjected to high temperatures or thermal cycling. MXenes can undergo structural transformations or phase transitions at elevated temperatures, leading to reduced stability and performance. Researchers are investigating methods to enhance the thermal stability of MXene-based soft actuators, such as introducing thermal barrier coatings or optimizing the MXene structure to withstand higher temperatures. MXenes are frequently employed in electrochemical appliances, namely energy storage devices or sensors. In these cases, stability against electrochemical reactions, such as oxidation or dissolution, is critical to ensure long-term performance. Additional explorations ought to focus on improving the electrochemical stability of MXene-based soft actuators by modifying the MXene surface, designing protective coatings, or exploring MXene composites with enhanced stability.

### Power Supply and Efficiency

Addressing power supply challenges, such as developing efficient energy storage and delivery systems, is a prerequisite to ensure reliable and sustainable operation [72, 73]. In a recent study, researchers unveiled a bioinspired MXene-centered bimorph actuator featuring an asymmetric incrusted microstructure. This innovative actuator demonstrated the remarkable ability to harness natural sunlight and attain directional self-propulsion [74]. Li et al. [75] introduced a novel smart soft actuator that possesses several remarkable capabilities, including humidity-driven actuation, self-powered humidity sensing, humidity energy harvesting, and instantaneous motion pursuing. This actuator was designed using an MXene/ polystyrene sulfonic acid/cellulose composite membrane as its foundation. Its unique mechanism relied on the asymmetric expansion caused by a moisture gradient, allowing it to convert the chemical potential of humidity into mechanical force [75]. One approach to creating self-powered MXene soft actuators involves the integration of MXene with other materials, including polymers or hydrogels. This hybrid design allows for the combination of MXene's electrical conductivity and mechanical flexibility with the desired properties of the additional materials. The ensuing actuators act in response to outside stimuli, such as temperature or humidity, and exhibit programmable shape-changing behavior [29]. MXene-based soft actuators can also harvest energy from their surrounding environment, enabling self-sustainability. By utilizing the piezoelectric or triboelectric properties of MXene, these actuators convert mechanical or thermal energy into electrical energy. This energy can then be deployed to power the actuator itself or other electronic devices, making them highly efficient and environmentally friendly. Due to their unique properties, MXene-based soft actuators can achieve autonomous motion without the need for external power sources. By utilizing stimuli-responsive materials and incorporating feedback mechanisms, these actuators can sense and respond to changes in their environment. This allows them to adapt and move in a controlled and precise manner, opening up possibilities for applications such as soft robotics and biomimetic systems.

### Integration of Multiple Functionalities

There are some limitations to the design of MXenes for bioinspired soft actuators with angle-independent structural color while incorporating additional functionalities. Developing methods to integrate sensing capabilities, self-healing properties, or other functionalities without sacrificing performance or color stability requires careful consideration and innovative design approaches [60, 76]. The development of innovative design approaches to integrate multiple functionalities into MXene-based soft actuators can be complex and requires cautious consideration of the actuator's structure, materials, and fabrication techniques. Notably, the introduction of additional functionalities to MXene-based soft actuators may lead to performance trade-offs. For instance, incorporating self-healing properties or sensing capabilities could affect the actuator's response time, mechanical strength, or actuation range. Consequently, striking the right balance between functionality and performance is a critical challenge that researchers need to address.

### Cost-effectiveness

MXenes, being relatively new materials, are expensive to produce in larger quantities. Balancing the cost-effectiveness of MXene-centered soft actuators with the desired functionalities and angle-independent structural color is must for practical applications and extensive implementation [77, 78]. Fine-tuning the manufacturing processes for MXene-based soft actuators be able to contribute to cost reduction while the automation, process optimization, and streamlined workflows can minimize the production time and labor costs. In addition, advancements in additive manufacturing or 3D printing techniques may offer cost-effective manufacturing options for complex actuator designs.

### Color-tunable Soft Actuators

A diverse range of soft actuators capable of tuning both color and shape deformations (or locomotion) have been developed, indicating potential for usages in domains like soft robotics and biomedical appliances [79]. For instance, bioinspired fluorescence color-tunable soft actuators have been developed that are endowed with self-healing properties, reconfigurable nature, and shape memory performance [80]. One of the challenges in the development of MXene-centered soft actuators with angle-independent structural colors lies in achieving color-tuning capabilities that rival the robustness of natural systems, such as chameleons. Presently, many color-tunable soft actuators exhibit monotonous colors and indistinguishable color changes, which fall short of the dynamic and vibrant color transformations witnessed in nature. Overcoming this hurdle requires a deeper understanding of the underlying mechanisms of natural color-changing systems and the development of innovative strategies to replicate and enhance these capabilities in MXene-based soft actuators. By bridging this gap, researchers can unshackle the full potential of MXene-centered soft actuators and realize their vision of achieving chameleon-like color-tuning capabilities in artificial materials.

### Biocompatibility and Toxicity Issues

Several studies have focused on assessment of biocompatibility of MXenes and their composites, which requires studying the interactions between MXenes and biological entities such as cells, tissues, and organs. It encompasses evaluating their cytotoxicity, hemocompatibility, immunological response, and overall biocompatibility profile. Several factors influence the biocompatibility of MXenes and their composites, including their surface chemistry, size, morphology, and the presence of functional groups [81–83]. Further studies are required to fully understand the interactions of MXenes and their derivatives with living systems. Assessing their long-term biocompatibility and potential cytotoxicity is essential when considering their use in biomedical applications. Functionalization can be employed to enhance the biocompatibility of MXenes, thus rendering them suitable for biomedical applications. By modifying the surface with biocompatible molecules or polymers, MXene-based soft actuators can be made compatible with living systems, thus reducing the risk of adverse reactions. The lack of standardized protocols for evaluating the biocompatibility of MXenes and their composites hinders the comparability and reproducibility of research findings. Establishing standardized procedures would facilitate accurate assessment and comparison of biocompatibility data. Most of the biocompatibility studies have aimed on short-term effects, while the long-term influences of MXenes and their composites on biological systems remain largely unexplored. Investigating their durability, stability, and potential accumulation in the body is crucial for assessing their long-term biocompatibility. Notably, current biocompatibility studies have typically focused on single-cell interactions, but understanding how MXenes and their composites interact with complex multicellular systems, such as tissues and organs, is essential for real-life applications. While MXenes and their composites show promise in laboratory-scale studies, challenges arise when scaling up production processes. Ensuring consistent quality, safety, and biocompatibility in large-scale manufacturing is a key obstacle to address. In this context, surface modification techniques can be employed to enhance the biocompatibility of MXenes and their composites. Functionalizing their surfaces with bioactive molecules or polymers can improve their interaction with biological systems and mitigate potential adverse effects. By optimizing the type, concentration, and distribution of MXenes in composite materials, researchers can tailor their properties to meet specific biocompatibility requirements. The employment of advanced characterization techniques, such as high-resolution imaging and spectroscopy, is capable of  providing deeper insights into the interactions between MXenes and biological systems. This can aid in understanding the underlying mechanisms of biocompatibility and guide the design of more biocompatible materials. Furthermore, extensive in vivo studies are needed to further assess the biocompatibility of MXenes and their composites, thus offering valuable information regarding their systemic effects, biodistribution, and potential long-term consequences. Establishment of a comprehensive database that consolidates biocompatibility and toxicity data for MXenes and their derivatives would be highly beneficial for researchers and industry professionals. Such a resource would facilitate knowledge sharing, data comparison, and accelerate the advancements in the fabrication of biocompatible materials.

Scientists are exploring ways to incorporate additional functionalities into MXene-based soft actuators, such as self-healing capabilities or multi-responsive behavior [84–86]. This would enable these actuators to adapt to changing environmental conditions and improve their overall performance. With additional explorations and technological advancements, one can anticipate the development of advanced functionalities, integration with other smart materials, energy harvesting capabilities, biomedical applications, multi-material fabrication techniques, and environmental applications. In addition, MXene-based soft actuators can be integrated with additional smart materials, namely shape memory polymers or conductive polymers, to fabricate hybrid systems with improved capabilities [87]. By combining the unique properties of MXenes with other functional materials, researchers can develop soft actuators that exhibit complex, programmable behaviors, such as shape morphing or temperature-responsive actuation. Furthermore, MXene-centered soft actuators have the capability to harvest energy from their surroundings, enabling self-powered and autonomous systems [88–90]. By integrating energy harvesting mechanisms, such as piezoelectric or triboelectric generators, into MXene-based soft actuators, researchers know how to create self-sustainable devices that can harvest energy from mechanical or environmental sources. MXenes are already being explored for biomedical appliances, like drug delivery schemes or tissue engineering scaffoldings [91–93]. In the future, MXene-based soft actuators could find even wider use in the biomedical field. By ensuring the biocompatibility and safety of MXenes, researchers can develop soft actuators that can be implanted or used in wearable devices for biomedical monitoring, rehabilitation, or assistive technologies. Currently, MXene-based soft actuators are primarily fabricated using MXene films or coatings. In the future, advancements in additive manufacturing techniques, namely 3D/4D printing, could facilitate the creation of intricate and customized MXene-based soft actuators. This would open up newer openings for the design and production of complicated and functional soft robotic systems. MXene-centered soft actuators could also have usages in environmental monitoring and remediation. By integrating sensing capabilities into these actuators, they can be deployed to detect pollutants or monitor environmental conditions. These MXene-centered soft actuators are capable of being utilized in soft robotics for underwater exploration or marine conservation efforts.

The future prospects of MXene-centered soft actuators with angle-independent structural colors are promising. These materials have the potential to contribute to various fields, including display technologies, camouflage systems, sensors, and beyond. By achieving angle-independent structural colors, MXene-based soft actuators can offer enhanced color perception and consistent coloration across different viewing angles, providing improved user experiences and expanding the possibilities for applications. One exciting prospect is their integration into wearable devices and smart textiles. These materials can be retained to produce dynamic and receptive color-changing fabrics, enabling innovative designs and functionalities in the fashion and textile industry. Imagine garments that are able to change color based on environmental conditions or user preferences, thus enhancing personal expression and interactivity. In the field of displays, MXene-centered soft actuators with angle-independent structural colors hold the potential to develop visual experiences. Traditional displays often suffer from limited color accuracy and degradation at various viewing angles. By utilizing MXene-based soft actuators, displays can achieve vibrant and consistent colors from all viewing perspectives, leading to more immersive and realistic visual displays. Furthermore, MXene-centered soft actuators with angle-independent structural colors be able to find applications in the development of advanced camouflage systems. By mimicking natural color-changing abilities, these materials enable adaptive camouflage that blends seamlessly with the surroundings, increasing stealth and security in military and defense applications. For sensing applications, these materials can enhance the sensitivity and accuracy of optical sensors. By incorporating these materials into sensor designs, it is possible to create sensors that are capable of detecting and responding to even subtle changes in color, leading to improved precision in a variety of sensing applications such as environmental monitoring, biomedical diagnostics, and industrial automation. However, additional explorations are needed to optimize the mechanical properties, durability, and reliability of these materials; the scalability and cost-effectiveness are important considerations for practical implementation in various applications.

## Conclusion

The ability to achieve angle-independent structural color in synthetic materials is a significant advantage. This property allows for vibrant and iridescent colors that remain consistent from different viewing angles, opening up possibilities for creating visually appealing and dynamic designs. MXene-based soft actuators exhibit excellent flexibility, making them suitable for applications where conformability and adaptability are mandatory. Their soft and pliable nature enables them to interact with delicate objects and navigate complex environments. These soft actuators demonstrate rapid actuation and recovery speeds, which is advantageous for dynamic movements and quick response times, thus empowering agile and responsive behavior in robotic systems. Overall, the future prospects of MXene-centered soft actuators with angle-independent structural colors are promising. These materials have the potential to improve various fields, offering enhanced color perception, adaptive camouflage, better quality display technologies, and advanced sensing capabilities. More elaborative studies in this area will pave the way for innovative applications, thus providing advanced dynamic and responsive materials.
